# β-Dispersion of blood during sedimentation

**DOI:** 10.1038/s41598-021-82171-x

**Published:** 2021-01-29

**Authors:** Ahmet C. Sabuncu, Sinan Muldur, Barbaros Cetin, O. Berk Usta, Nadine Aubry

**Affiliations:** 1 Mechanical Engineering Department, Worcester Polytechnic Institute, Worcester, MA 01609 USA; 2 Center for Engineering in Medicine, Massachusetts General Hospital, Harvard Medical School and Shriners Hospitals for Children, Boston, MA 02114 USA; 3 Microfluidics & Lab-On-a-Chip Research Group, Department of Mechanical Engineering, I.D. Bilkent University, Ankara, Turkey; 4 School of Engineering, Tufts University, Medford, MA 02155 USA

**Keywords:** Biomedical engineering, Electrical and electronic engineering

## Abstract

Aggregation of human red blood cells (RBC) is central to various pathological conditions from bacterial infections to cancer. When left at low shear conditions or at hemostasis, RBCs form aggregates, which resemble stacks of coins, known as ‘rouleaux’. We experimentally examined the interfacial dielectric dispersion of aggregating RBCs. Hetastarch, an RBC aggregation agent, is used to mimic conditions leading to aggregation. Hetastrach concentration is incrementally increased in blood from healthy donors to measure the sensitivity of the technique. Time lapse electrical impedance measurements were conducted as red blood cells form rouleaux and sediment in a PDMS chamber. Theoretical modeling was used for obtaining complex permittivity of an effective single red blood cell aggregate at various concentrations of hetastarch. Time response of red blood cells’ impedance was also studied to parametrize the time evolution of impedance data. Single aggregate permittivity at the onset of aggregation, evolution of interfacial dispersion parameters, and sedimentation kinetics allowed us to distinguish differential aggregation in blood.

## Introduction

Human red blood cells (RBCs) have tendency to form aggregates at low shear or hemostasis conditions by joining face-to-face^1^. These aggregates, which resembles stacks of coins, are known as ‘rouleaux’. There are a few important features of these structures. First, unlike blood coagulation, in which RBCs and platelets are enmeshed in fibrinogen strands, RBC aggregation is a reversible process. Rouleaux can break down into smaller structures or individual RBCs under high shear conditions. Two main factors affecting RBC aggregation is, first, RBC suspending medium, and second, RBC surface charge density, both of which may change with pathological conditions. For example, RBC aggregation was shown to be enhanced in patients with acute bacterial infection^2^, patients with morbid obesity^3^, patients with normal tension glaucoma^4^, and subjects with sickle cell trait^5^. Both plasmatic and cellular factors can affect RBC aggregation, for example, correlations were shown between plasma concentrations of C-reactive protein and fibrinogen versus RBC aggregation in bacterial infection^2^, and fibrinogen versus RBC aggregation in experimental sepsis^6^. Moreover, a reduction in RBC surface charge was observed during experimental sepsis^6^. Other factors that influence RBC aggregation include temperature^7^, RBC membrane viscoelasticity, and cytoplasm viscosity^8^.

The golden clinical standard to determine RBC aggregation is to measure erythrocyte sedimentation rate using the Westergren technique. In this technique, blood is let to sediment in a vertically mounted tube, which is optically transparent for visual observation. After an hour of sedimentation, supernatant level is measured visually. This inexpensive technique reports a non-specific parameter in mm/h that is a function of RBC sedimentation. This parameter is known as Erythrocyte Sedimentation Rate (ESR). In the course of one hour, RBCs not only aggregate to form linear rouleaux, but also form networks. Eventually these networks collapse. Pribush et al. showed that when RBCs are let to aggregate first rouleaux form by having RBCs join face to face, which is followed by formation of branched rouleaux that have face to side RBC connections^9^. As RBCs settle further, these branched rouleaux undergo macrostructural reorganization with cell-free zones propagate from bottom towards the surface^10^. Finally, this network of RBCs collapse at a much shorter time scale, and fragments of this network settle toward chamber bottom^11^. Therefore, the evolution of RBC aggregates starts with formation of linear rouleaux, followed by branched network of rouleaux by face to side connections, compression and collapse of the RBC network, and finally sedimentation of the fragments. All of these process have their unique time scales, and the main determinants of the ESR is the compression of the network and the settling of the network fragments^11^. The ‘delay time’ between the formation of rouleaux and the collapse of the network could range from few minutes to an hour, and this delay time is a function of the hematocrit value and the strength of the RBC aggregation^12^.

Other techniques have been also developed to measure ESR. The main motivation is mainly to shorten the measurement time and minimize the required sample volume (1 ml). For instance, in syllectometry, light is incident on a layer of blood that is subject to an initial shear stress to break existing rouleaux in blood. The scattered light, then, is parametrized to assess RBC aggregation as a function of time^13^. Electrical impedance measurements of blood is also utilized for determining the sedimentation rate of RBCs. In these measurements, a pair of electrodes records impedance of a blood sample as RBCs aggregate and sediment, and a correlation is made between impedance features and the sedimentation rate. For instance, correlations are shown between the sedimentation rate and equivalent circuit parameters extracted from impedance measurements conducted at three frequencies^14^, resistance at 50 kHz^15^, impedance at 200 kHz^16^, DC conductivity^17^, and relaxation frequency^18^.

When a material is placed in an alternating electric field, two effects are evident. First is an ohmic current. Second is that charges inside materials separate and align with the electric field, forming dipoles. Dipoles can undergo a ‘relaxation’ process if the frequency of the alternating electric field is altered. Blood, in an alternating electric field, exhibits four dielectric relaxation mechanisms, which are α, β, δ, and γ dispersions^19^. While α dispersion is due to the polarization of counterion cloud in the electrical double layer around a cell, existence of α dispersion for RBC suspensions is doubtful and a recent study did not observe α dispersion for blood^20^. β dispersion, also known as interfacial dispersion, is due to charging of the cell membrane. γ dispersion arises from reorientation of dipolar water molecules in an alternating electric field, and this dispersion in blood is different than that in pure water due to the presence of macromolecules, such as proteins^20^. Schwan introduced δ dispersion as a weak dispersion between β and γ dispersions, and ascribed this process to the polarization of polar sub-groups or bound water to hemoglobin^19^. Most electrical studies on the RBC sedimentation rate fell under β dispersion that has a relaxation frequency around 1 MHz and is a strong function of RBC volume fraction, RBC size and cell membrane capacitance.

While studies investigating the electrical impedance of blood during RBC aggregation presented a correlation between some impedance features, such as impedance at a given frequency, and sedimentation rate, the entirety of the β dispersion is not studied during RBC aggregation. Studies that measured impedance at a specific frequency to measure RBC aggregation only captures a small amount of information in the entire plethora of RBC polarization and relaxation. Often, approximations and assumptions needs to be introduced in such measurements. The corresponding approximations are that (i) plasma is the main conducting pathway for ohmic current and (ii) capacitance have not experienced any effects due to β-relaxation when measurements are conducted at low frequencies, which are below the assumed β-relaxation frequency. However, capturing the entire spectrum of impedance, especially for the β-dispersion can reveal a breadth of data, enabling one to use mixture theories and shell models for biological cells^21^. For instance, the accurate asymptotic behavior of blood at low frequencies could be calculated from the measured spectrum. As physical properties of the suspended phase may be extracted exclusively from these physical models, the use of such models may also enable elimination of the electrical effects of differential RBC volume fraction and plasma properties among different blood samples. Therefore, one could obtain dielectric properties of sedimenting RBCs using physical theories of dielectric relaxation independent of the suspending medium and the volume fraction. Furthermore, the knowledge of the dielectric spectrum a priori also allows mitigation of the electrode polarization effect after the extraction of the impedance data^22^. A disadvantage of this technique is that the instrumentation, which can perform a wide-frequency impedance measurement are relatively expensive, and as an alternative, the available low-cost integrated circuits can only perform a narrow frequency sweep^23^.

Various physical models have been proposed to simulate impedance of individual RBCs and suspension of RBCs in media. These models are summarized in a recent textbook^21^. Briefly, models consider RBCs biconcave shape. Previous studies modeled RBCs as oblate spheroids^24–26^, and experimental studies with animal^27^ and human^28^ RBC have confirmed the validity of the oblate spheroid model. The ellipsoidal model entails two distinct β dispersion processes^25^. These two processes are related to the relaxation along major and the minor axes of the spheroid. However currently, there are no cell and mixture models that can simulate dielectric spectrum of linear or branched rouleaux. An optimum choice, therefore, could be a single shell model with an effective equivalent cell radius for aggregates, as in^10^, and Hanai’s mixture model that considers particle–particle interactions at higher volume fractions of suspended cells^29^ for a cell model and a mixture theory, respectively.

In the present study, we investigated the β dispersion of differentially aggregating blood in a wide frequency range, from 1 kHz to 100 MHz. A specific focus is on the initial aggregation regime, the first few minutes of the aggregation, sufficiently long before the collapse of the RBC network. We characterized this dielectric response using the Cole–Cole model and mixture theories that allows for a focus on the dielectric response of RBC cell membrane. The dielectric response of blood samples with modulated RBC aggregation rates are compared. These aggregation rates in blood from healthy donors are tuned with hetastarch, a well-known RBC aggregation agent. The specificity of the dielectric response of blood in determining these aggregation rates is investigated.

## Materials and methods

### RBC sedimentation

Sedimentation is due to density variations in the constituent phases of a medium in the presence of an external field. At steady sedimentation velocity, the force due to the gravitation field and the density differential is balanced by the Stokes’ drag at low Reynolds number regime. The terminal sedimentation velocity of a single RBC (${v}_{RBC}$) treating RBC as a spherical particle is therefore given by,

1 $${v}_{Stokes}=\frac{2}{9}\frac{\left({\rho }_{RBC}-{\rho }_{plasma}\right) {{R}_{RBC}}^{2} g}{\eta }$$

where $g$ is the gravitational constant, $\rho$ is the density, ${R}_{RBC}$ is the equivalent radius, and $\eta$ is the dynamic viscosity of the plasma. Considering the sedimentation of RBCs in whole blood, there are limitation in using Stokes’ drag to model RBC sedimentation. Therefore, the following modifications were made to the terminal velocity given by Eq. (1). We followed the study by Oka^30^ for altering the terminal velocity. First, since Stokes’ law is valid for rigid solid particles, the radius in Eq. (1) needs to be modified to account for the biconcave shape of RBCs. Accordingly, the hydrodynamic drag force on a sedimenting discoid body with radius $a$ is equal to that of a sphere of radius $ka$. Here, the constant $k$ depends on the orientation of the discoid during sedimentation. In the work of Oka^30^, this constant was 0.71 that was based on microscopic observations of RBC sedimentation. Therefore, for a single RBC, we used an effective radius (${R}_{o}$) that is equal to $0.71a$, where $a$ is radius of the biconcave disc. Second, since the Stoke’s drag is valid for an isolated particle, the Stoke’s velocity needs to be corrected for a dense suspension of particles. A semi-empirical correlation proposed by Richardson and Zaki^31^ is implemented as:

2 $${\nu \mathord{\left/ {\vphantom {\nu {\nu _{{Stokes}} }}} \right. \kern-\nulldelimiterspace} {\nu _{{Stokes}} }} = (1 - \varphi )^{m}$$

where *φ* *is volume fraction and* $m$ is an empirical exponent, specifically 4.65 for low Reynolds number particulate flows.

Finally, the effective radius of a single RBC needs to be modified to account for the aggregating RBCs. In whole blood under hemostasis, there will be a distribution of RBC sizes, depending on the number of cells in a rouleau. Following the recent work by Zhbanov and Yang^32^, the following expression is used for the radius of aggregated particles:

3 $${R}_{agg}\left(t\right)= {R}_{o}+ {\nu }_{agg}{\int }_{0}^{t}\frac{1}{(1-\varphi )}exp\left[-{\left(\frac{{t}^{*}}{{t}_{agg}}\right)}^{2}\right]d{t}^{*}$$

where ${\nu }_{agg}$ and ${t}_{agg}$ are empirical coefficients characterizing the rate of aggregate formation [m/s] and the characteristic time of aggregation, respectively.

Although several different approaches are available in the literature for the dynamical modeling of RBC sedimentation, a simplified approach is implemented^32^. The blood column is divided into N equal thin layers, and a constant velocity is assigned for each thin layer during a time step (assuming each time step is sufficiently small). Following a continuity equation for each layer, one can write:

4 $${\varphi }_{j}\Delta {z}_{j}= constant$$

The velocity of the top surface of layer $j$ is defined as ${v}_{j}$, and it is obtained using Eq. (2) in which the terminal velocity, ${v}_{Stokes}$, can be obtained using Eq. (1), with the effective radius being replaced by the radius of the aggregated cells following Eq. (3). The final set of equations represents a nonlinear set of first order differential equations which can be integrated by the implicit Euler method. Once the RBC concentration profile (${\varphi }_{i}$) is known, the impedance at each layer could be computed using mixture models, which will be discussed in the forthcoming sections. The total impedance then is the serial combination of these impedance elements.

### Blood samples

All experiments were performed in accordance with relevant guidelines and regulations. The study was approved by Northeastern University Office of Environment Health & Safety and Institutional Review Board prior to the experiments. Human whole blood was purchased from Innovative Research (Novi, MI). Received samples were unidentified, and included only age, sex, and race information of the donor. The samples were tested negative for HIV Hepatitis B and C, Syphilis, and Zika virus prior to experiments. Na Heparin was used as an anti-coagulant. Hematocrit values are reported by the company for each sample. Experiments reported in this study were conducted as soon as samples were received in a box that was kept at 4 °C. Samples that were stored more than one week at 4 °C exhibited significant changes in the dielectric spectra. Experiments are performed in at least four independent replications. Plasma of the blood samples was separated by centrifuging whole blood at 10,000 rpm for 10 min in a microcentrifuge (Eppendorf, Hamburg, Germany). Some measurements required resuspension of blood cells in Phosphate Buffered Saline (PBS). For those measurements, RBCs was washed with PBS prior to the experiments. Experiments were conducted at room temperature (21.1 °C).

In order to simulate differential amounts of RBC aggregation in a blood sample, we used HetaSep (STEMCELL Technologies Inc., Cambridge, MA), which is an erythrocyte aggregation agent to separate nucleated cells from RBC. Four different samples differential amounts of HetaSep were prepared. Table 1 summarizes specific amounts of Hetasep in each sample. As blood samples were prepared according to the content in Table 1, the samples were mixed gently with a pipette, and loaded into the PDMS chamber. Impedance experiments were, therefore, conducted with a few seconds delay after loading the measurement chamber.

**Table 1:** Blood samples used in experiments to determine RBC aggregation.

Sample 1	840 μl blood + 0 μl HetaSep + 160 μl PBS	–
Sample 2	840 μl blood + 40 μl HetaSep + 120 μl PBS	0.28% (wt/v) HES
Sample 3	840 μl blood + 80 μl HetaSep + 80 μl PBS	0.57% (wt/v) HES
Sample 4	840 μl blood + 160 μl HetaSep	1.14% (wt/v) HES

The active ingredient of HetaSep is 6% (wt/v) Hetastarch (HES), which is a synthetic, nonionic hydroxyethyl derivative of starch used as a plasma expander. Its recommended concentration to initiate rouleaux formation in blood is 1:5 (HES/Blood, Sample 4 represents approximately this ratio)^33^. It is also crucial to mention the aggregation mechanism induced by HES. At hemostasis, the RBC surface charge produces a repulsive force to maintain the stability of the suspension. As summarized by Baskurt et al.^1^, there are two theories to explain RBC attraction. In the bridging theory, macromolecules in the extracellular space adsorb onto the RBC surface, and link two adjacent cells together^34^. The depletion theory assumes a local osmotic pressure increase near the RBC surface because of the concentration difference between the cell surface and the bulk medium^35^. This results in flow of water from cell surface to the medium, and therefore, an attractive force between RBCs. There is some evidence that RBC aggregation due to HES is because of bridging interactions^36^. The same study reported a decreased electrophoretic mobility for RBCs treated with HES.

### Impedance measurements

**Figure 1:**
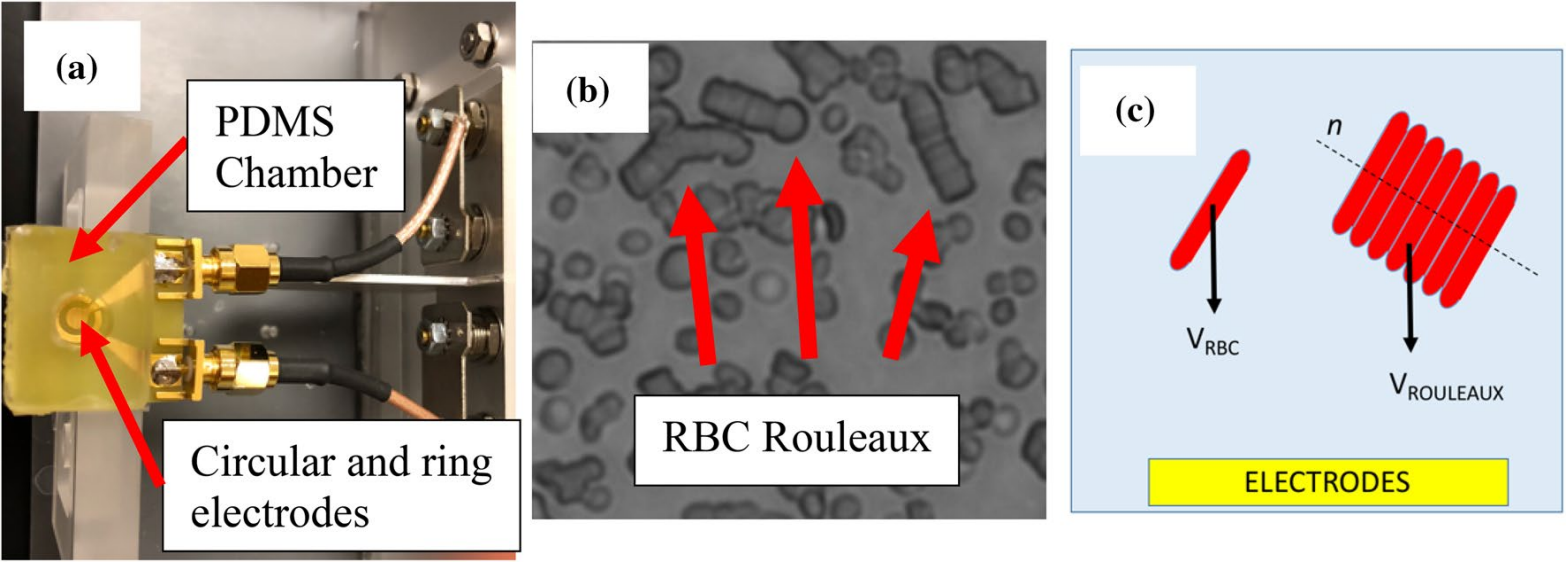
Blood samples were housed in a PDMS chamber and their electrical impedance was measured using circular and ring electrodes as shown in (**a**). As blood is left at hemostasis in autologous plasma, RBCs aggregate to form ‘rouleaux’. A representative microscopic picture of RBC rouleaux is shown in (**b**). As the sedimentation velocity of a particle in low Reynolds number regime is dependent on its radius squared, RBC Rouleaux sediment faster than RBCs. A schematic depicting the cross-section of the chamber is shown in (**c**).

We used a planar coaxial sensor (golden parts in Fig. 1a, circular and ring electrodes in the picture constitutes the coaxial sensor) to measure the electrical impedance of the blood sample between 1 kHz and 100 MHz using Keysight E4990A. The electrodes are nanostructured by sintering 80 nm gold nanoparticles (Sigma-Aldrich Corp, St. Louis, MO) to reduce the undesired effects of the electrode polarization (EP) as described in a previous study^37^. A polydimethylsiloxane (PDMS) chamber is made to house the blood sample. The circular opening on the PDMS was 7 mm. In order to determine the optimum amount of blood, the chamber was loaded with increasing amounts of PBS using increments of 50 μl (please see Fig. S1). As a result, we used 200 μl of blood sample in the experiments.

The impedance analyzer was controlled using MATLAB (The Mathworks Inc., Natick, MA) over local area network. The impedance data were acquired over the 1 kHz—100 MHz range for every 2.2 s until 256 timepoints. The methodology to obtain dielectric property spectra of the biological samples was explained in a previous study^38^. Here, a brief description is given for continuity. The sample admittance is given using the following relationship,

5 $${Y}_{sample}=k\left({\sigma }_{sample}+j\omega {\varepsilon }_{sample}^{^{\prime}}{\varepsilon }_{o}\right)$$

where $${\varepsilon }_{sample}^{^{\prime}}$$ is the relative permittivity of the sample, $${\sigma }_{sample}$$ is the conductivity of the sample, $${\varepsilon }_{o}$$ is vacuum permittivity, and $$k$$ is cell constant, respectively. $${\sigma }_{sample}$$ is composed of DC conductivity and frequency dependent dielectric loss term. Measured impedance ($${Z}_{measured})$$ is affected by the blood sample and as well as the EP. In this study, in order to model the measured impedance, we used the constant phase element model (CPE) to represent the EP impedance, and the Cole–Cole dielectric relaxation model to represent the sample impedance. According to this approximation, the impedance takes the expression,

6 $${Z}_{model}={\left(j\omega k{\varepsilon }_{model}^{*}\right)}^{-1}={\left[{\left(j\omega /{\omega }_{0}\right)}^{n}\Lambda {\varepsilon }_{o}\right]}^{-1}+{\left[j\omega {C}_{o}\left({\varepsilon }_{h}+\frac{{\varepsilon }_{l}-{\varepsilon }_{h}}{1+{\left(j\omega \tau \right)}^{a}}-\frac{j{\sigma }_{DC}}{\omega {\varepsilon }_{o}}\right)\right]}^{-1}$$

where ${\varepsilon }_{model}^{*}$ is for the complex permittivity of the model, ${\omega }_{0}=1 rad/s$, $\Lambda$ is the CPE coefficient, $n$ is the CPE exponent, ${C}_{o}$ is the capacitance of the ‘empty capacitor’ that is equal to $k{\varepsilon }_{0}$, ${\varepsilon }_{l}$ and ${\varepsilon }_{h}$ are the low and high frequency limits of the suspension relative permittivity, respectively, $\tau$ is the dielectric relaxation time constant, $a$ is a parameter that accounts for the broadening of the dispersion and varies between 0 and 1, and ${\sigma }_{DC}$ is the DC (effective) conductivity. The ‘cell constant’ $k$ in Eq. (6) is measured using dielectric fluids such as air and deionized water. A fitting algorithm using the nested *lsqnonlin* function in MATLAB (2016a, Mathworks) was used to find the optimum model parameters that minimized the residual, which is given as:

7 $${\chi }^{2}=\sum_{i}{\left(1-log{\varepsilon }_{sample,i}^{^{\prime}}/log{\varepsilon }_{model,i}^{^{\prime}}\right)}^{2}+\sum_{i}{\left(1-{\sigma }_{sample,i}/{\sigma }_{model,i}\right)}^{2}$$

In the above equation, index i represents each of the 101 data points between 1 kHz and 100 MHz. The residual in Eq. (7) involved the logarithm of the relative permittivity terms because these are at least three orders of magnitude larger than the conductivity terms. Using the above models and the fitting procedure, the experimental data could now be expressed in terms of the Cole–Cole parameters ${\varepsilon }_{l}, {\varepsilon }_{h},\uptau , a,$ and ${\sigma }_{DC}$.

Once the dielectric spectra of blood is known, one can use a mixture theory to find the complex permittivity of cells. In doing so, the effects of external medium and volume fraction of cells could be ruled out from the experimental data. For concentrated cell suspensions, Hanai’s mixture model could be used to calculate the cell complex permittivity (${\varepsilon }_{c}^{*}$) from the suspension complex permittivity (${\varepsilon }_{sus}^{*}$)^29^,

8 $$1 - \varphi = \left( {\frac{{\varepsilon _{{sus}}^{*} - \varepsilon _{c}^{*} }}{{\varepsilon _{{med}}^{*} - \varepsilon _{c}^{*} }}} \right)\left( {\frac{{\varepsilon _{{med}}^{*} }}{{\varepsilon _{{sus}}^{*} }}} \right)^{{{1 \mathord{\left/ {\vphantom {1 3}} \right. \kern-\nulldelimiterspace} 3}}}$$

where the subscript $med$ stands for the external medium. Separate measurements were made to determine the dielectric properties of the suspending media in a single experiment. The following quantities are used for relative permittivity and DC conductivity of plasma mixed with HES: 72.4 and 1.12 S/m. The change of these quantities between plasma and various concentrations of HES was negligible as the variations were less than 1%. PBS relative permittivity and DC conductivity is 71.9 and 1.46 S/m. Once Cole–Cole parameters are known, ${\varepsilon }_{sus}^{*}$ is calculated, and later using Eq. (8), the single cell complex permittivity is calculated.

### Low frequency behavior of blood

The interfacial polarization of blood is a function of dielectric properties of its constituents: cellular structures and plasma. Dielectric studies of blood considered only plasma and RBC properties, since RBCs constitute more than 99% of cells by volume^19^. The Cole–Cole model is used to represent β dispersion of blood. A correlation between the Cole–Cole parameters and physical properties of RBCs could be made using Hanai’s mixture equation (Eq. 8) and the single shell model for biological cells assuming the following approximations: the membrane conductivity is negligibly small compared with the conductivity of the cell interior (by a factor of $\sim {10}^{-5}$,^39^); the membrane thickness is negligibly smaller than the cell radius (the cell membrane is in $nm$ range while RBCs are in $\mu m$ range); the low frequency conductivity of cells is negligibly smaller than that of the external medium as the cell membrane is made mostly of poorly conducting lipids. As a result, the low frequency dielectric behavior of blood is^40^,

9 $${\sigma }_{DC}= {\sigma }_{med}{\left(1-\varphi \right)}^\frac{3}{2}$$

10 $${\varepsilon }_{l}={\varepsilon }_{med}{\left(1-\varphi \right)}^\frac{3}{2}+\frac{3}{2}\left\{1-{\left(1-\varphi \right)}^\frac{3}{2}\right\}{\varepsilon }_{c,0}$$

where subscripts $c,$ and $0$ stand for cell and low frequency limit, respectively, and ${\sigma }_{DC}$ is the conductivity at DC. In the above equation, ${\varepsilon }_{c,0}$ is the low frequency permittivity of an RBC, and using the above approximations, it takes the following form^41^,

11 $${\varepsilon }_{c,0}=\frac{{C}_{mem} r}{{\varepsilon }_{v}}$$

where ${C}_{mem}$ is the membrane capacitance, $r$ is the equivalent cell radius, and ${\varepsilon }_{v}$ is vacuum permittivity.

### Statistical analysis

We used One-way ANOVA with Tukey’s post-hoc test for multiple comparison of values obtained for different samples (S1–4) and negative control (PBS). Shapiro–Wilk test was used to test normality. Differences between means were considered significant at p < 0.05. We present the results as box plots. The top of the box represents the mean and the standard deviation is shown with a bar. Data points are represented with dots. Probability values are indicated on figures by asterisks, as follows: *p < 0.05; **p < 0.01; ***p < 0.001; ****p < 0.0001. Graphing and statistical tests were performed using Prism 8 software (GraphPad Software Inc.).

## Results and discussion

### Time response of dispersion parameters

**Figure 2:**
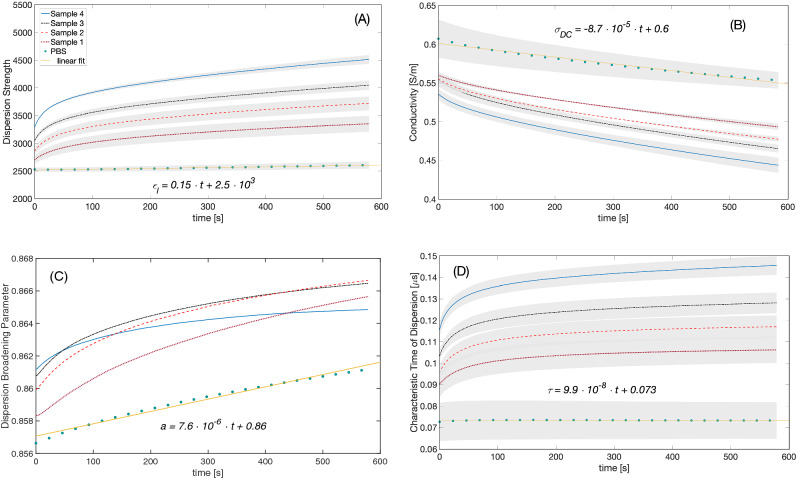
Dispersion strength (${\varepsilon }_{l}-{\varepsilon }_{h}$) (**a**), conductivity (${\sigma }_{DC}$) (b), dispersion broadening parameter ($a$) (**c**), and dielectric relaxation time constant ($\tau$) (**d**) of the blood samples. Different color lines correspond to different samples. Mean data from experiments with hematocrit values 42%, 43%, and 47% are shown. The shaded area around each curve represents the standard error. The standard error is not shown for the dispersion broadening parameter for clarity. The inset equations are linear fits to PBS data.

The dielectric spectra of four blood samples with changing amounts of RBC aggregation agent as well as whole blood and blood cells suspended in PBS were recorded in time series in independent experiments. For each time point, the data was fitted to the dielectric dispersion model given by Eq. (6), which produced the Cole–Cole parameters. This process resulted in 5120 (4 experiments × 5 samples × 256 data points) dielectric spectra and the same number of sets of the model parameters. A representative dielectric spectrum and the model fitted to the measured data are shown in the supporting information (Fig. S2). The β (interfacial) dispersion of the blood sample around 1 MHz is evident from this figure. While the dielectric model we used was a close fit to the data, the model resulted in a relatively small error, and L2 Norm of this error is given in the supporting information for a representative experiment (Fig. S3). Here, we report the following Cole–Cole parameters, the dispersion strength (${\varepsilon }_{l}-{\varepsilon }_{h}$), the DC conductivity (${\sigma }_{DC}$), the dispersion broadening parameter ($a$), and the dielectric relaxation time constant ($\tau$) for the interfacial dispersion in blood. Figure 2 summarizes the change of the mean model parameters with respect to time. In all samples except for RBC in PBS, an exponential growth or decay was observed for all parameters. For the RBC in PBS sample, the parameters evolved linearly with time, except for $\tau$, which was constant. Parameters $\tau$ and ${\sigma }_{DC}$ exhibits a relatively weak non-linear behavior for the first ~ 40 s of the experiments. In addition, the parameter $\tau$ was relatively constant for the PBS suspension. In addition, according to Fig. 2, at time 0, the samples had distinct Cole–Cole parameters, which suggests that within seconds before the measurement could be conducted HetaSep creates aggregation in blood.

We next discuss the results in Fig. 2. The linear characteristic of RBC sedimentation in saline suspensions is well known. In the absence of plasma proteins, RBC aggregation diminishes, and therefore, the RBC sedimentation velocity is constant in PBS, leading to linear changes in dielectric spectra as evident in Fig. 2 (for example, please see work of Oka^30^). We observed an exponential growth in the dispersion strength as a function of sedimentation in samples with HES. Equation (10) suggest that the low frequency permittivity is a function of a single RBC’s low frequency permittivity (please see Fig. S4). The low frequency permittivity behavior of an RBC is linearly proportional to its size and its membrane capacitance (Eq. 11). As RBCs aggregate and form rouleaux, the cell radius in Eq. (11) is replaced with an effective aggregate size and the cell membrane capacitance is altered to reflect the aggregate morphology. In addition, the concentration of RBC rouleaux increase at the bottom of our measurement chamber where the electric field lines are concentrated near the coaxial sensor. As a result, with increased RBC aggregation increases in ${\varepsilon }_{c,0}$ and the dispersion strength are expected. This statement is also validated with a finite difference model of RBC rouleaux^42^. According to this numerical study, for random orientation of RBC rouleaux, aggregation increases the low frequency permittivity of RBC suspension. Furthermore, in this computational study by Asami and Sekine^42^, the characteristic time for the dielectric dispersion increases with the increasing number of RBCs in a rouleaux whose direction is parallel to the measuring electric field. In this study, a similar observation is made in Fig. 2d. Broadening of the dielectric dispersion is attributed to a distribution of relaxation times in a biological system^43^. Cell size distribution, for instance, makes the dispersion broadening parameter ($a$) larger; therefore, the increase of the broadening parameter in Fig. 2c could indicate an increasingly wider size distribution in blood. Independent size experiments are required to verify this correlation.

### Empirical model for low frequency permittivity time data

We evaluated time changes in the low frequency permittivity (${\varepsilon }_{l}$) data as this data showed the aggregation effects of varying HES concentration in plasma clearly. It is evident in Fig. 2a that time characteristics of ${\varepsilon }_{l}(t)$ are also different among the samples. The kinetics of RBC sedimentation affects ${\varepsilon }_{l}(t)$ of blood, and therefore, one could infer about the RBC aggregation kinetics by investigating the time dependence of ${\varepsilon }_{l}\left(t\right)$. This is also evident in Eq. (10), where ${\varepsilon }_{l}$ is expressed as a function of the RBC parameters and volume fraction. For this purpose, the data corresponding to experiments with samples 1–4 are represented with a double exponential model.

12 $${\varepsilon }_{l}\left(t\right){=A e}^{\frac{-t}{{\tau }_{1}}}+ {B e}^{\frac{-t}{{\tau }_{2}}}+\mathrm{C}$$

where $A,B, C, {\tau }_{1},$ and ${\tau }_{2}$ are constants. The sum $A+B+C$ is the low-frequency permittivity at the initial time ${\varepsilon }_{l}(0)$. As discussed earlier, low frequency permittivity data of RBCs suspended in PBS is best represented using a linear model. Experimental data is fit to the double exponential model using a nonlinear least squares model. The model was an excellent fit to the experimental data with coefficient of determination (R^2^) values greater than 0.99. A sample fit to experimental data is shown in the supporting information (Fig. S5).

**Figure 3:**
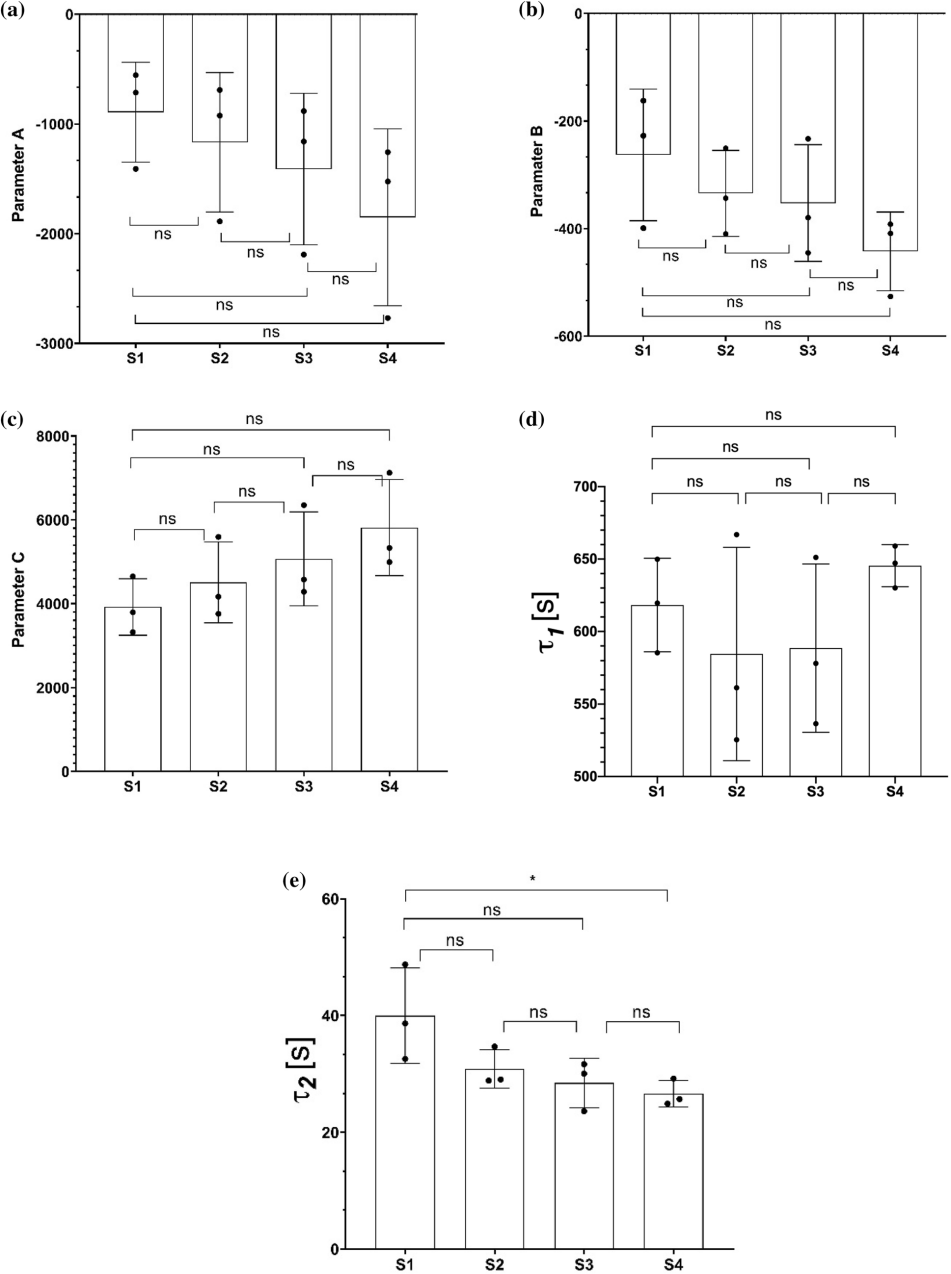
Emprical model parameters ($A$ (**a**), $B$ (**b**), $C$ (**c**), $, {\tau }_{1}$ (**d**), and ${\tau }_{2}$ (**e**). The data is computed using the experimental data from measurements and Eq. (11). Results are shown using box plots. One-way ANOVA with Tukey’s post-hoc test is used for multiple comparison. Probability values are indicated on figures by asterisks, as follows: *p < 0.05; **p < 0.01.

We compared the parameters of this empirical model for RBC sedimentation for varying HES concentration. The parameters are shown using box plots in Fig. 3. The empirical two exponential model suggests two processes with different time scales. The first time constant, ${\tau }_{1}$, suggested a process characterized with 600 s, while the second constant, ${\tau }_{2}$, suggested a process characterized with 30 s. According to the results, while mean ${\tau }_{2}$ is significantly different between the samples, the only significant change was between samples 1 and 4 (p < 0.05) as evident in Fig. 3. The other parameters ($A$, $B$, $C$,${\tau }_{1}$) did not return any significant changes with changing HES. It is possible that HES induces a relatively faster aggregation that involves formation of linear rouleau from individual RBCs. On the other hand, ${\tau }_{1}$ could be related to the formation of larger structures such as 3-D networks of rouleaux as RBCs make face to side connections^9^, which is not affected by HES according to the present data. Further experiments are required to understand this process. Similar double exponential models are also used to parametrize the aggregation kinetics in syllectograms^44^. In syllectometry, the smaller time constant is attributed to the formation of rouleaux and the larger time constant in the double exponential function is related to the three-dimensional aggregation process in which rouleaux form RBC networks^13^.

### Comparison of dispersion parameters at ${t}=100\,{s}$

**Figure 4:**
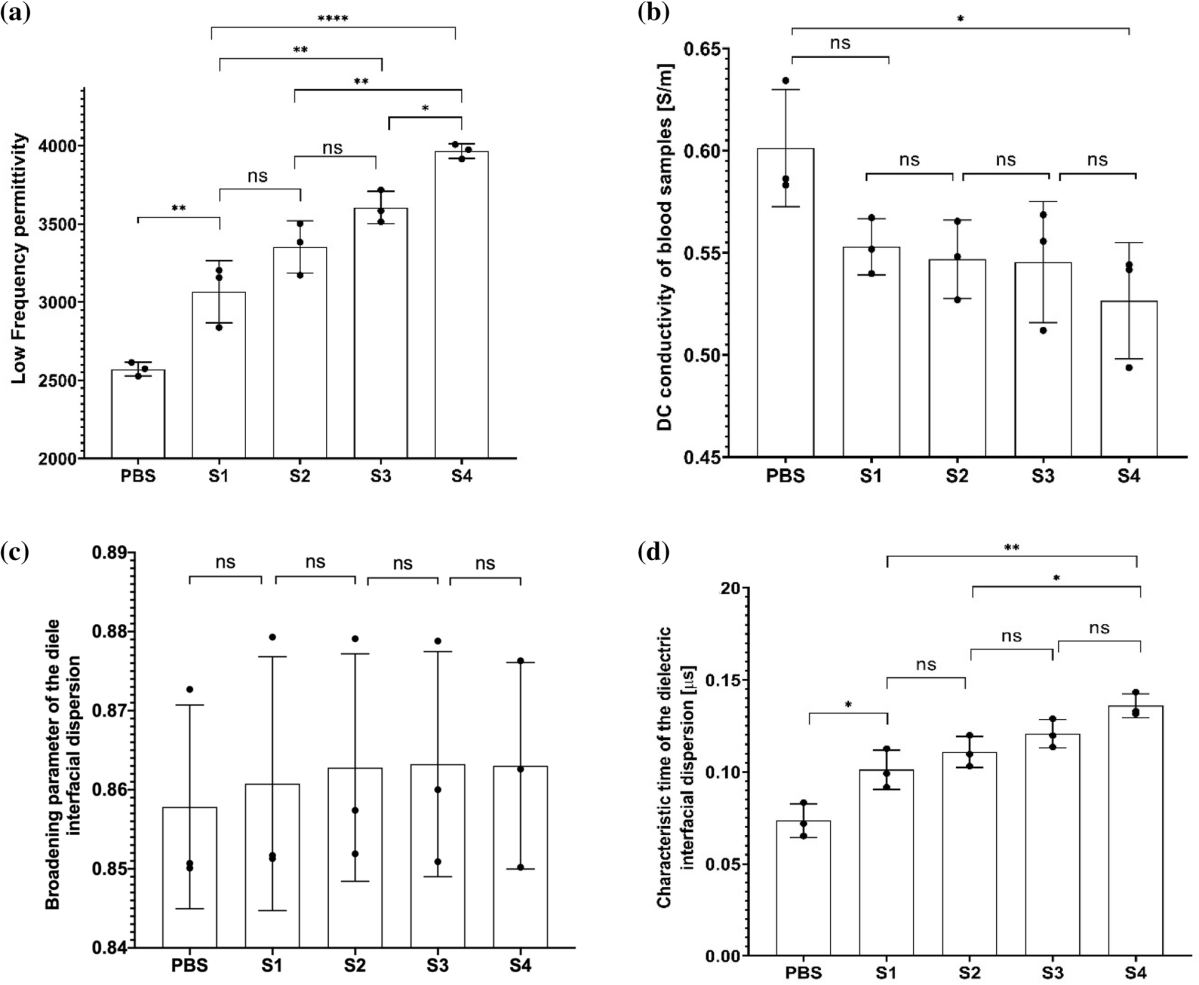
Comparison of low frequency permittivity (${\varepsilon }_{l}$) (**a**), conductivity (${\sigma }_{DC}$) (**b**), dispersion broadening parameter ($a$) (**c**), and dielectric relaxation time constant ($\tau$) (**d**) of the experimental data from measurements with blood samples of varying HES concentration. The displayed data is for time point $t=100 s$. Results are shown using box plots. One-way ANOVA with Tukey’s post-hoc test is used for multiple comparison. Probability values are indicated on figures by asterisks, as follows: *p < 0.05; **p < 0.01, ***p < 0.001, ****p < 0.0001.

As the time required to obtain results from a diagnostic test is an important parameter for practical reasons, we ideally would like to determine the aggregation characteristics of RBC in as short time scales as possible. To make this possible, we searched for the timepoint which yields the maximum differences in Cole–Cole parameters. The maximum difference occurred for times larger than $\approx 2 {\tau }_{2}$ (please see the supporting information, Fig. S6). We investigated the effects of aggregation agent HES on Cole–Cole parameters at $t=100 s$ that is slightly larger than $2 {\tau }_{2}$. The comparison of the Cole–Cole parameters against HES concentration is given in Fig. 4. While the change from negative control (PBS) to the lowest concentration of HES is significant for ${\varepsilon }_{l}, {\sigma }_{DC}$ and $\tau$ data, we did not observe significant changes for ${\varepsilon }_{l}$ and $a$. Changing HES concentration from sample 1 to sample 4 yielded significant changes in ${\varepsilon }_{l}$ and $\tau$. The smallest HES concentration change we could resolve was 0.57% (wt/v) (for S1 to S3, p < 0.01), and this was using the ${\varepsilon }_{l}$ data. The changes in the dispersion broadening parameter $a$, did not achieve significance for changing concentration of HES.

### Modeling low frequency permittivity response at ${t}=0\,{s}$

**Figure 5:**
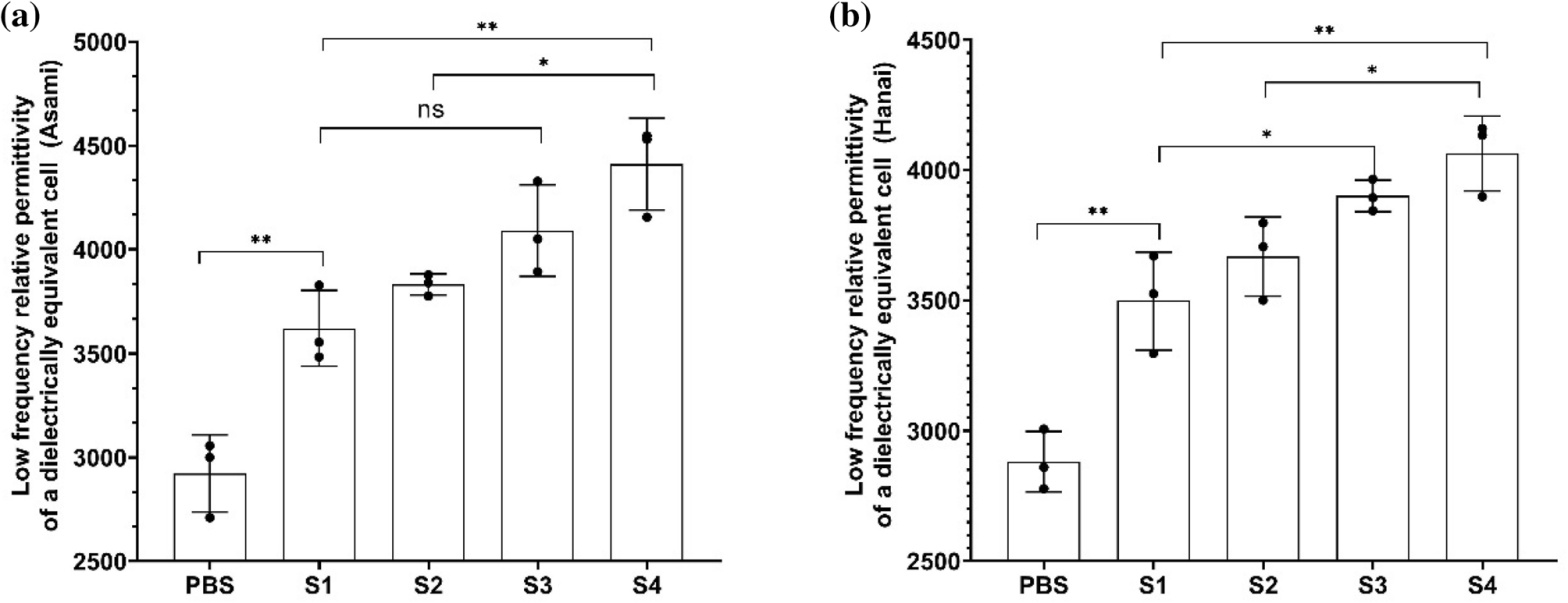
Comparison of ${\varepsilon }_{c,0}$ calculated using Asami’s approximation (**a**) and Hanai’s model (**b**). The data is computed using the experimental data from measurements with blood samples of varying HES concentration. Results are shown using box plots. One-way ANOVA with Tukey’s post-hoc test is used for multiple comparison. Probability values are indicated on figures by asterisks, as follows: *p < 0.05; **p < 0.01.

The low frequency permittivity data ${\varepsilon }_{l}$, is investigated in detail as it can further be decomposed into complex permittivity of cells (${\varepsilon }_{c}$), using Eqs. (8) and (10). Ideally, one would like to see a negligible scatter of the dispersion strength between independent measurements conducted using the same amount of HES. Therefore, effects of external medium (plasma) and volume fraction should be ruled out from the measured data using a theoretical model. However, an analytical approach that could solve for the complex permittivity of an RBC considering heterogeneous distribution and biconcave shape at high volume fraction does not exist. While numerical simulations of an individual rouleau with finite difference schemes exist, they are currently not practical in modeling the experimental data^45,46^. As RBCs aggregate and sediment towards the bottom of the chamber, the RBC concentration in top layers decrease, and concentration increase in bottom layers. Therefore, the volume fraction of cells across the chamber will not be uniform during sedimentation. However, assuming a uniform volume fraction in the measurement chamber for the initial time point ($t=0 s$), one could calculate cell complex permittivity, ruling out the volume fraction. We tested two different approaches for calculating ${\varepsilon }_{c}$ in this study. For both of these approaches, we assumed an average dielectric radius for modeling the dielectric behavior of aggregating RBCs suspensions. Accordingly, under this assumption, samples would involve the homogeneous distribution of spherical particles of radius $r$ that will produce the same dielectric behavior as aggregating RBC suspensions would produce at the same volume fraction. We used Hanai’s mixture model^29^ and Asami’s approximation^40^ – Eqs. (8) and (10), respectively – for the calculation of ${\varepsilon }_{c}$. These models require knowledge of the medium properties (${\varepsilon }_{sus}^{*}$), and medium dielectric properties are measured in separate experiments. The pairwise comparison of the resulting cell permittivity values at the low frequency limit (${\varepsilon }_{c,0}$) with varying HES is given in Fig. 5a and 5b, respectively, for Asami’s approximation and Hanai’s mixture model. While analysis results with Hanai’s model could resolve changes from S1 to S3 (0.57% HES change) with p < 0.05, the approach with the Asami’s model could not resolve the same change. We could resolve the same change using the ${\varepsilon }_{l}$ data at $t=100 s$ with p < 0.01. Measurement results obtained at $t=100 s$, could therefore resolve the HES change with a narrower confidence interval.

### Estimation of aggregation parameters using the analytical model

**Figure 6:**
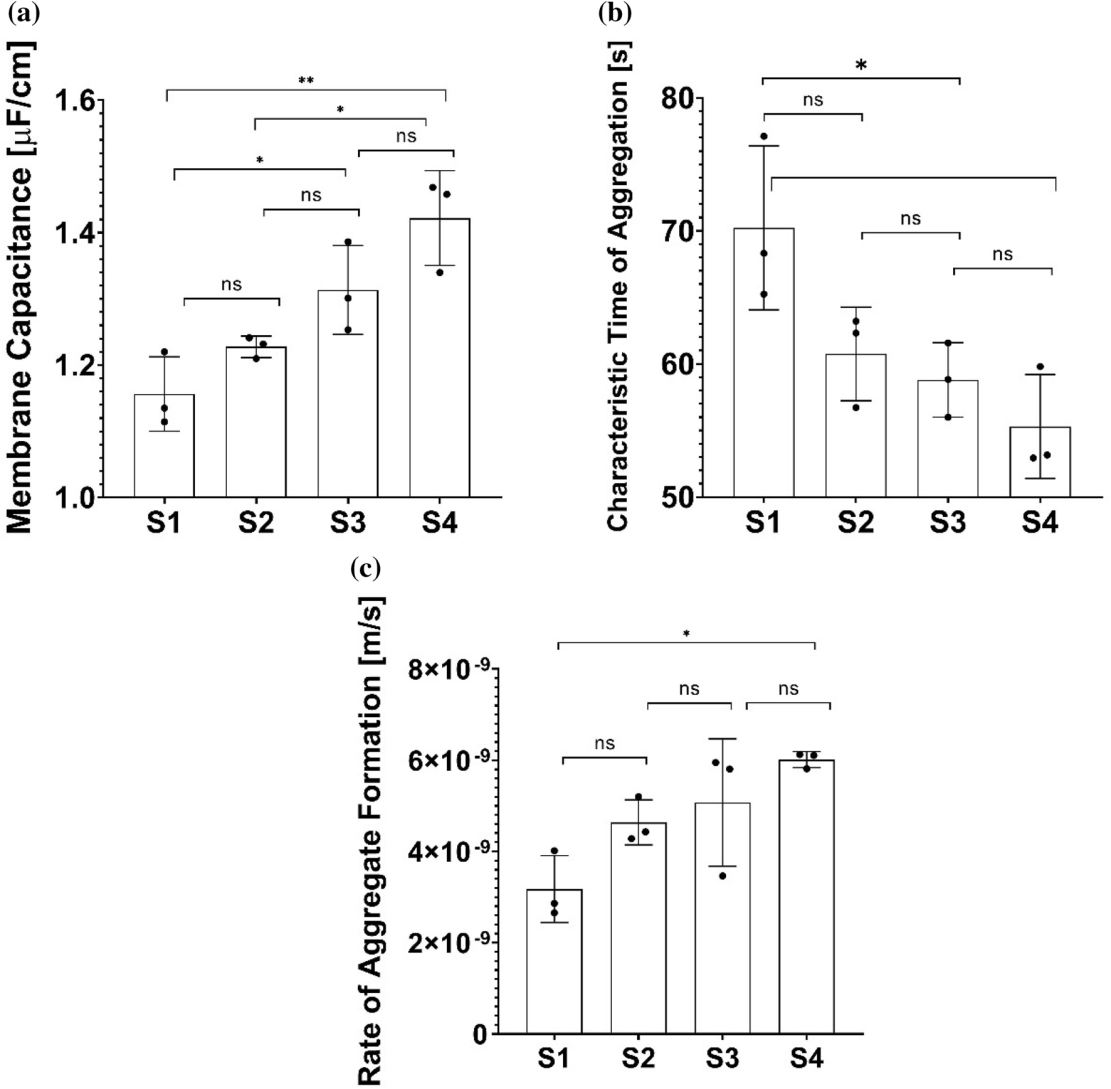
Analytical model parameters (${C}_{mem}$ (**a**), ${t}_{agg}$ (**b**), ${\nu }_{agg}$ (**c**)). The parameters are estimated using the ${\varepsilon }_{l}\left(t\right)$ data until time point $t=100 s.$ Results are shown using box plots. One-way ANOVA with Tukey’s post-hoc test is used for multiple comparison. Probability values are indicated on figures by asterisks, as follows: *p < 0.05; **p < 0.01.

Next, we employed the continuity equation presented earlier to find aggregation parameters ${\nu }_{agg}$, ${t}_{agg}$, and membrane capacitance ${C}_{mem}$, to model the time response of aggregating RBCs. We first solve the continuity equation to find the volume fraction in each layer, ${\varphi }_{i},$ in the blood column. Once the volume fraction is known, low frequency admittance of each layer is found using Eq. (10) and cell constant, $k$. The impedance response of blood is then equal to serial combination of each layer’s impedance. Because the model in Eq. (3) involves only one time constant, the time response until time point $t=100 s$ is modeled. While using this approach one could relate RBC physicochemical properties to RBC sedimentation process, the model is not trivial and one needs nonlinear regression to find the best combination of ${\nu }_{agg}$, ${t}_{agg}$, and ${C}_{mem}$ to represent the low frequency permittivity time response, ${\varepsilon }_{l}\left(t\right)$. Parameters ${C}_{mem}, {\nu }_{agg}$ and ${t}_{agg}$ can be utilized to gauge the RBC sedimentation kinetics. The comparison of these parameters for different samples is given in Fig. 6. While the membrane capacitance is close to 1 $\mu F/cm$ that is a widely-accepted value for an unfolded, smooth cell membrane and the time constant is close to the values of ${\tau }_{2}$ given in Fig. 3e. According to the results in Fig. 6, ${C}_{mem}$ and ${t}_{agg}$ yields significant changes between S1 and S3, that corresponds to 0.57% (wt/v) HES change. Both ${t}_{agg}$, and ${C}_{mem}$ also exhibited significant changes between sample 1 and sample 4.

A similar numerical approach by Zhbanov and Yang^32^ could represent the time response of conductivity response even at larger time scales, it is clear that modifications in the RBC sedimentation theory were required so that reliable sedimentation parameters could be obtained. In these modeling efforts, one needs to consider the sensor geometry and a physical theory of sedimentation, such as the model in the work of Jung et al.^46^, which are both outside the scope of this work. The present study approximates the sample impedance such that each layer in the measurement chamber is serially combined. The present study also considered isotropic aggregation per Eqs. (3), (10) and (11), and the rationale for this assumption was that current dielectric mixture models could not account for the anisotropic structure of the rouleaux. While the present approach produced reasonable results, improved models could yield a superior resolution of the RBC aggregation. Therefore, there is an immediate need for mixture models that could account for the anisotropy in RBC aggregation. Such models could benefit from finite element modeling and could resolve different stages of RBC aggregation, from the formation of individual rouleaux to that of 3D networks of rouleaux.

## Conclusions

We demonstrated the use of impedance spectroscopy to measure sedimentation characteristics of aggregating RBCs. Aggregation agent HES is used to model aggregation at changing concentrations. The kinetics of RBC sedimentation are investigated. The present technique could resolve 0.57% (wt/v) HES change in plasma background using both data at $t=0 s$ and $t=100 s$ or studying the entire time response until $600 s$. At $t=0 s$ cell complex permittivity at low frequency limit using Hanai’s model exhibited significant changes with 0.57% HES change (p < 0.05). At $t=0 s$, the same HES change could be resolved with p < 0.05 only studying the low frequency limit of suspension permittivity. Modeling the entire time response until $600 s$ with a physical theory of sedimentation to obtain an effective electrically equivalent cell membrane capacitance and the rate of aggregate formation resolved 0.57% HES change with p < 0.05. All other dispersion parameters studied did not resolve this change in HES. The current physical theories for modeling dielectric response of rouleaux is limited; therefore, there is a need to develop a parametric model of rouleaux dielectric response in blood. This model could furthermore be interfaced with a physical theory of RBC sedimentation. Such models could report RBC surface potential or other related physicochemical properties of RBCs that would be an accurate way to characterize RBC’s ability to aggregate. From clinical perspective, aggregation during inflammatory diseases versus infections could be studied in order to better understand the RBC physicochemical properties in these different pathological states. Another venue of future research is to combine the present approach with other modalities, such as video microscopy and dynamic light scattering to increase the number of independent measurements. One such study is by Isiksacan et al.^47^, which measured the light intensity while also providing microscopic images during RBC aggregation.

## Supplementary Information

Supplementary Information.
