# Supplementary material for: β-Dispersion of blood during sedimentation
Source: Sci Rep. 2021 Jan 29;11:2642. doi: 10.1038/s41598-021-82171-x (PMC7846779; doi:10.1038/s41598-021-82171-x)
Supplement: Supplementary file 1 — Supplementary Information. [file 41598_2021_82171_MOESM1_ESM.pdf]

β-Dispersion of Blood during Sedimentation

Ahmet C. Sabuncu[1,*], Sinan Muldur[2], Barbaros Cetin[3], O. Berk Usta[2], Nadine Aubry[4]

Supplementary Information

Figure S1. Capacitance of different amounts of phosphate buffered saline was measured to determine the optimum amount of liquid in the chamber.

Figure S2. Measured (markers) and modelled (continuous line) dielectric spectra from a representative measurement (HCT=47% and sample 1, time point 2).

Figure S3. Square root of sum of residuals squared (L2 norm error) from a representative experiment with HCT = 47%.

Figure S4. Low frequency permittivity (ε_l) of the blood samples. Different color lines correspond to different samples

Figure S5. Time response of low frequency permittivity (ε_l) from a representative experiment with HCT = 45% and with conditions for sample 1 in Table 1.

Figure S6. The derivative of the difference between samples' low frequency permittivity (A) and dielectric relaxation time constant (B).

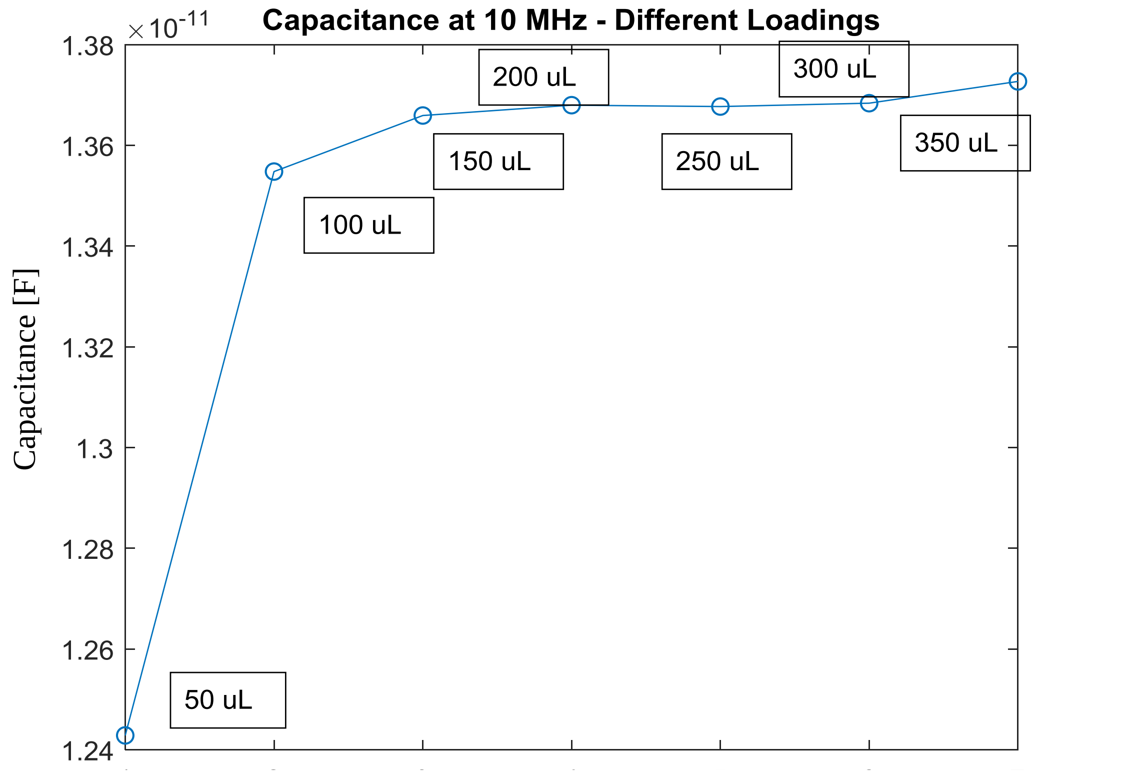

Figure S1. Capacitance of different amounts of phosphate buffered saline was measured to determine the optimum amount of liquid in the chamber.

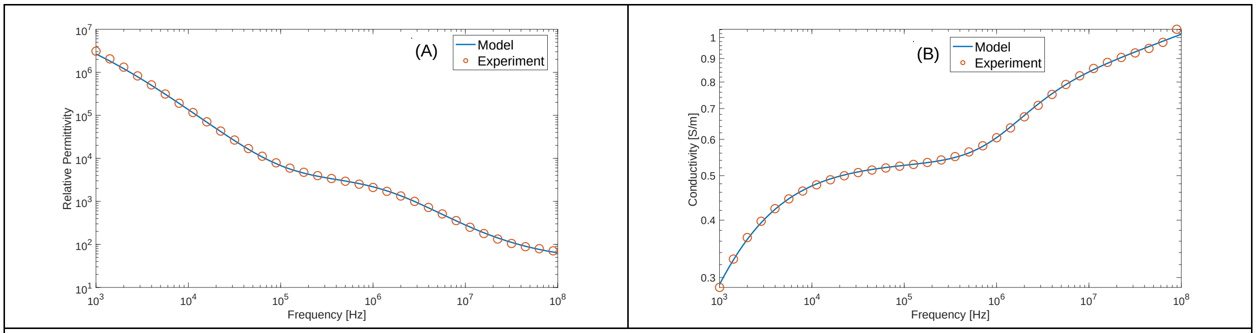

Figure S2. Measured (markers) and modelled (continuous line) dielectric spectra from a representative measurement (HCT=47% and sample 1, time point 2). Experimental data is plotted at every three frequency point for clarity. In figures (A) and (B) the relative permittivity and the conductivity spectra are shown, respectively.

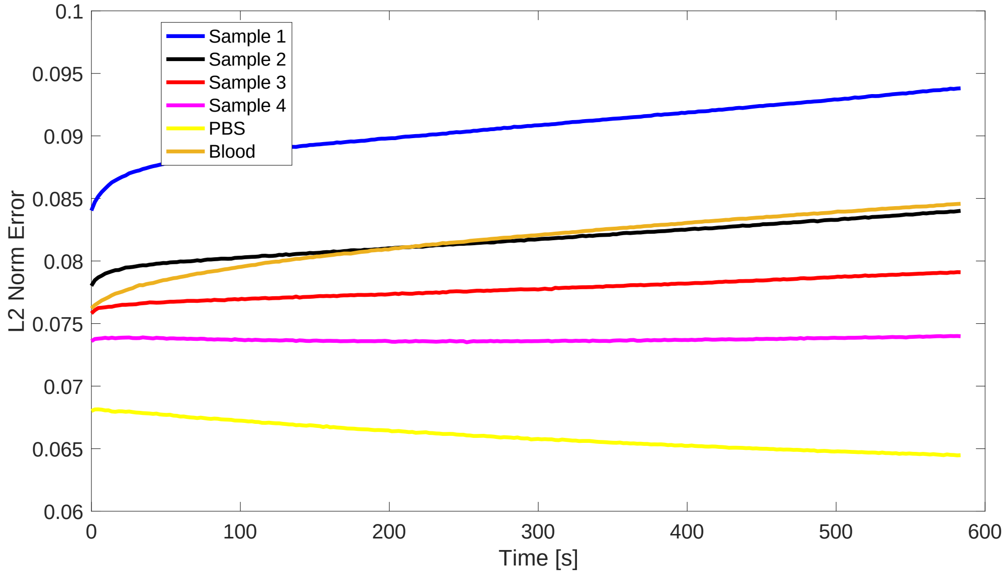

Figure S3. Square root of sum of residuals squared (L2 norm error) from a representative experiment with HCT = 47%.

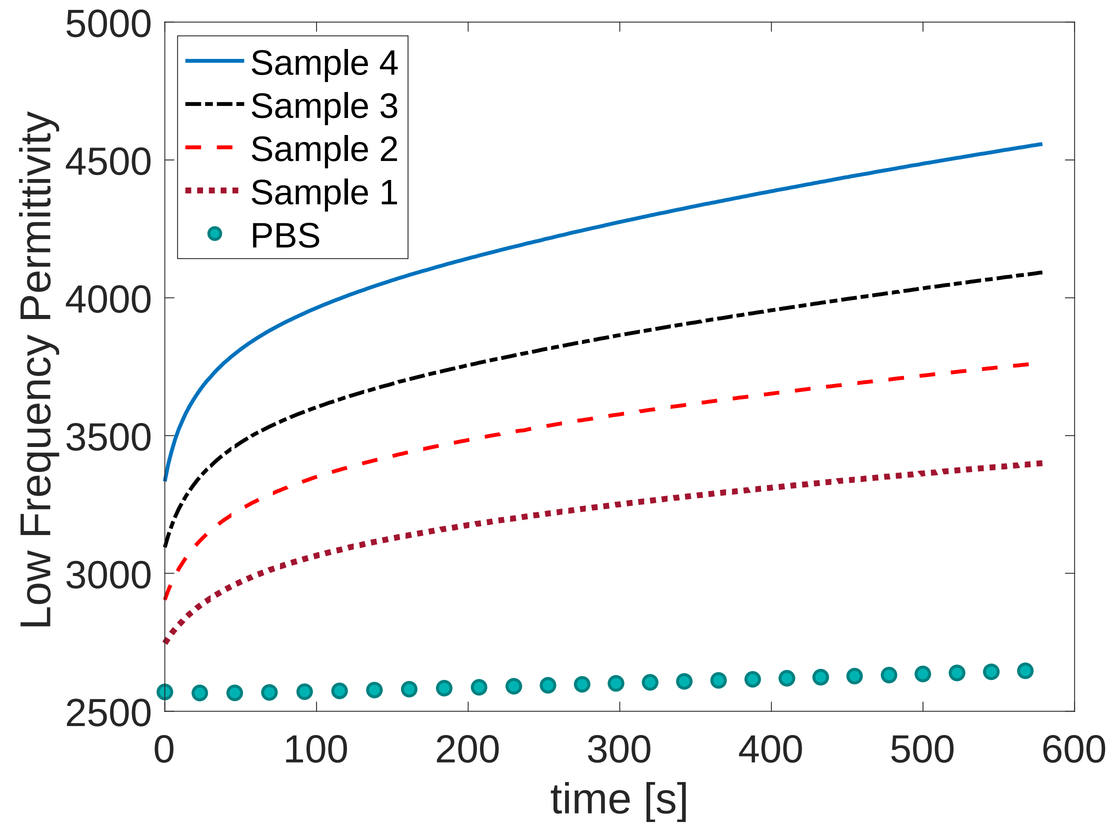

Figure S4. Low frequency permittivity (ε_l) of the blood samples. Different color lines correspond to different samples. Mean data from experiments with hematocrit values 42%, 43%, and 47% are shown. The shaded area around each curve represents the standard error.

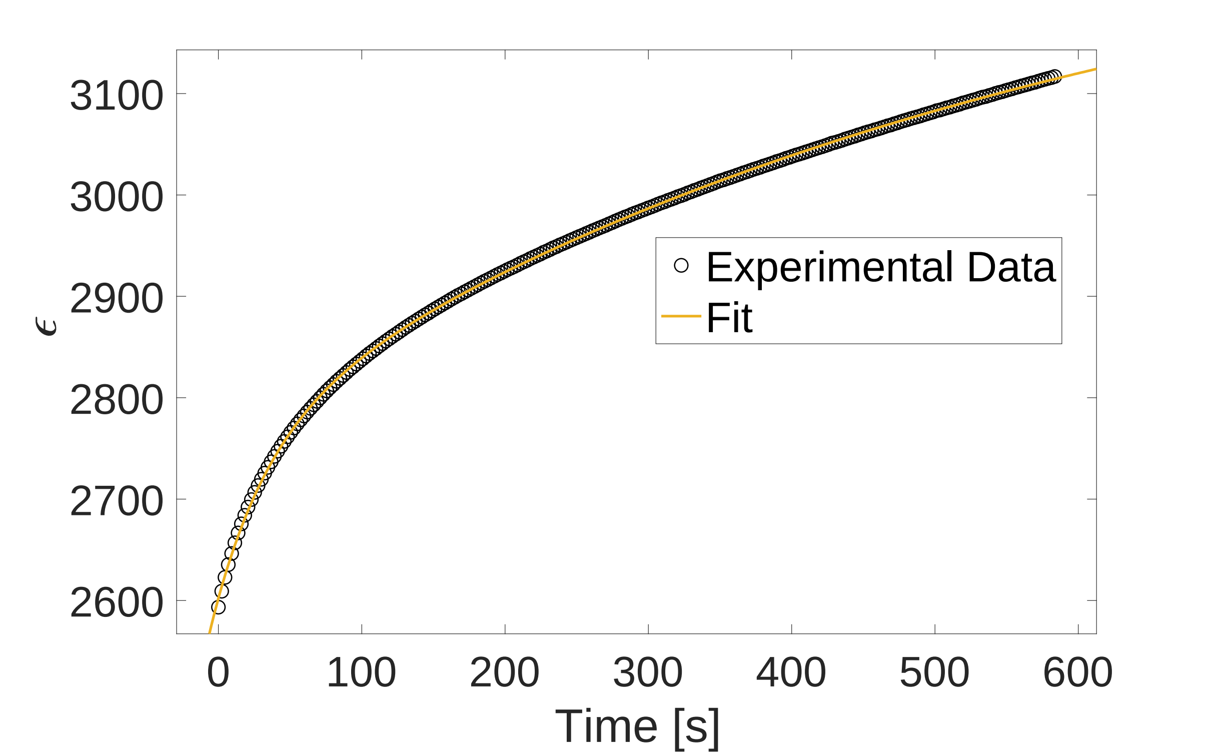

Figure S5. Time response of low frequency permittivity (ε_l) from a representative experiment with HCT = 45% and with conditions for sample 1 in Table 1. Black circles are the experimental data and the continuous line represents the model fit. R-square for this fit is 0.9999.

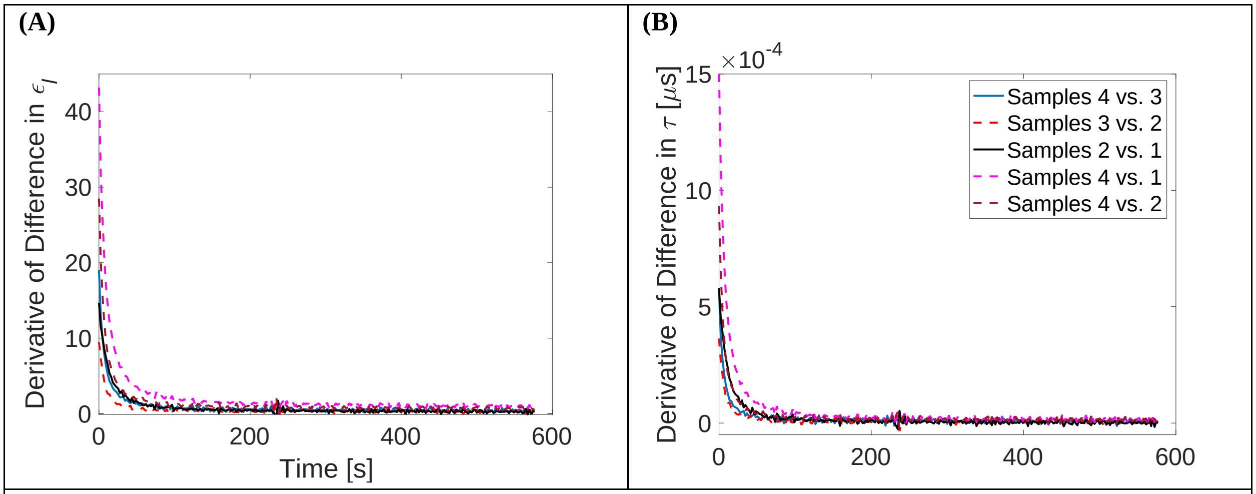

Figure S6. The derivative of the difference between samples' low frequency permittivity (A) and dielectric relaxation time constant (B).
